# Functional implications of the microbial community structure of undefined mesophilic starter cultures

**DOI:** 10.1186/1475-2859-13-S1-S2

**Published:** 2014-08-29

**Authors:** Eddy J Smid, Oylum Erkus, Maciej Spus, Judith CM Wolkers-Rooijackers, Svetlana Alexeeva, Michiel Kleerebezem

**Affiliations:** 1 Wageningen University, Laboratory of Food Microbiology, Bornse Weilanden 9, PO Box 17, 6700 AA, Wageningen, The Netherlands; 2 Top Institute Food and Nutrition (TIFN), P.O. Box 557, 6700 AN Wageningen, The Netherlands; 3 NIZO food research BV, P.O. Box 20, 6710 BA Ede, The Netherlands; 4 Wageningen University, Host Microbe Interactomics Group, De Elst 1, 6708 WD Wageningen, The Netherlands

## Abstract

This review describes the recent advances made in the studies of the microbial community of complex and undefined cheese starter cultures. We report on work related to the composition of the cultures at the level of genetic lineages, on the presence and activity of bacteriophages and on the population dynamics during cheese making and during starter culture propagation. Furthermore, the link between starter composition and starter functionality will be discussed. Finally, recent advances in predictive metabolic modelling of the multi-strain cultures will be discussed in the context of microbe-microbe interactions.

## Introduction

For modern, industrialised cheese production, two different types of starter cultures are being used: defined starter cultures and complex undefined starter cultures. Defined cheese starter cultures are usually composed of one or more strains with known characteristics. The individual strains in defined starter cultures, mostly mesophilic lactococci, usually were and still are being isolated from undefined complex starter cultures to obtain single strain cultures. The currently used undefined starter cultures originate from cheese production plants and cheese farms and were kept frozen to retain their original composition. Cultures used in the Netherlands for the production of Gouda cheese, like "Ur", "Fr8" and "Fr18" [1,2], have a long history of use [3] in a dairy environment and can be considered as domesticated cultures. From the moment these cultures were isolated in the 50's and 60's of the 20^th ^century, care has been taken to limit compositional shifts. This is achieved by minimizing the numbers of propagation cycles and by keeping sufficient aliquots of the frozen culture.

A key difference between defined starter cultures and undefined complex starters is their sensitivity to bacteriophage attack. In general, defined starters are more vulnerable to bacteriophage attack than undefined complex starters during production of cheese [1]. This feature as well as their function as a source for the isolation of new dairy strains with interesting functional characteristics, explains the renewed interest in undefined complex starter cultures [4]. The actual presence of bacteriophages in fully functional undefined complex starter cultures like "Ur" [1,2], may play a role in the inherent phage resistance of these cultures [3].

Microbiological research performed from the 70's to the 90's of the 20^th ^century demonstrated that complex Gouda starter cultures are dominated by *Lactococcus lactis *and sometimes contain *Leuconostoc mesenteroides *at lower abundance [5]. Furthermore, different functional variants and subspecies of *L. lactis *were identified. For instance citrate fermenting *L. lactis *subsp. *lactis *biovar. *diacetylactis *as well as caseinolytic and non- caseinolytic variants of *L. lactis *subsp. *cremoris *[6] could be distinguished. All these variants are thought to have a specific functional contribution to the process of cheese manufacturing. However, the actual complexity and diversity of undefined cheese starter cultures that goes beyond the sub-species discrimination remains obscure. With the advances made in high-throughput DNA sequencing technology and the substantially improved analytical capacities to quantitatively determine complex mixtures of metabolites, new opportunities are emerging to investigate structure and function of complex microbial communities that are applied in an industrial setting [4]. This review describes the recent advances made in studies of the microbial community of undefined complex cheese starter cultures. We report on work related to the composition of the cultures at the level of genetic lineages and representative strain-specific genome sequences, on the presence and activity of bacteriophages and on the population dynamics during starter culture propagation and cheese making. Furthermore, the correlation between starter culture composition and its functionality in cheese production will be addressed. Finally, the potential of predictive metabolic modelling of the multi-strain cultures will be discussed in the context of microbe-microbe interactions.

### Composition of undefined complex cheese starters

Traditional food fermentation processes have always been based on spontaneous fermentation driven by the development of the microbiota that naturally reside in the fermentable raw material [7]. The outcome of such processes is unpredictable because the inoculum is determined by the composition and microbial load of the fermentable raw material. The technique of back-slopping [8] can be seen as the earliest improvement of such spontaneous processes. With back-slopping, the fermentable raw material is inoculated with a small portion of a previously performed successful fermentation. This practice increases the success rate of natural fermentations and at the same time allows the microbial communities to develop towards an optimal composition. When repeated over many years, and provided that samples are not shared among producers, back-slopping allows for the natural evolution of independent microbial ecosystems [9]. The composition of a culture propagated by back-slopping in a (non-sterile) production environment, is a resultant of the continuously co-occurring processes of population dynamics, strain evolution and import of new biological material. Although not well documented, undefined complex starter cultures like "Ur" currently used in industrial Gouda cheese manufacturing [2] have their roots in artisanal cheese making at farm level. As such, the current microbial composition of these cultures is shaped by evolutionary forces and population dynamics driving towards optimal utilisation of raw milk. Although milk is, in terms of nutritional value, one of richest media on Earth, it is remarkable that complex cultures in general only contain two species of lactic acid bacteria (LAB), with dominant abundance levels of *L. lactis *and smaller communities of *Le. mesenteroides*. Apparently, the fast lactose fermenting LAB's create a monopolistic environment that prevents the establishment of other microbes in the community. The lactococci in these cultures can be quantified and characterised using classical microbial methods like the enumeration of colony forming units (CFUs) using various plating media that enable the detection of specific functional capacities per colony obtained. For example, the classical Reddy's agar medium [10] allows for simultaneously differentiation of *L. lactis *subsp. *cremoris *(no arginine deiminase activity, no citrate utilization), *L. lactis *subsp. *lactis *(arginine deiminase activity, no citrate utilization) and *L. lactis *subsp. *lactis *biovar. *diacetylactis *(arginine deiminase activity, citrate utilization). In parallel, the *Le. mesenteroides *populations can be enumerated on selective MRS plates [11], supplemented with vancomycin. The subspecies differentiation and nomenclature of the species *L. lactis *is based on rRNA gene restriction analysis that generally matches with the presence and absence of industrially relevant phenotypic traits [12]. Many different DNA-fingerprinting techniques have been developed that allow the differentiation of the genotypes of the two main subspecies, *L. lactis *subsp. *cremoris *and *L. lactis *subsp. *lactis *[13,14]. In a recent review paper, Ndoye and co-workers [15] describe currently available molecular tools that can be employed to characterize the microbiota in the cheese matrix.

**Figure 1:**
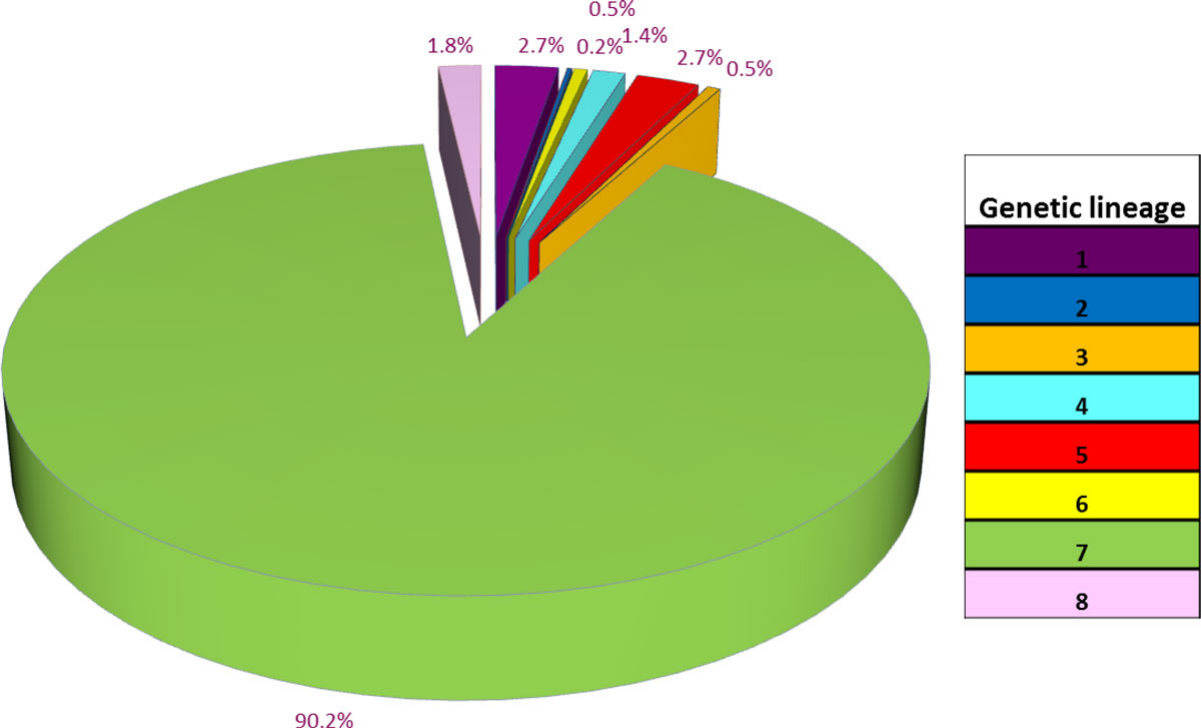
**Composition of undefined starter Ur**. The culture consists of *Lactococcus lactis *(lineages 1 to 7) and *Leuconostoc mesenteroides *(lineage 8). Calculation of the composition was based on data taken from [4].

Having in mind the above mentioned level of knowledge, Erkus and co-workers [4] set-out to design a research-blueprint for in-depth analysis of the microbial community of undefined Gouda cheese starters. This approach included the analysis of the genetic diversity among the community members beyond the level of subspecies. Initially, metagenome sequencing of the undefined starter culture was used to demonstrate the expected dominance of the species *L. lactis *and the smaller community contribution of the species *Le. mesenteroides*. Notably, these analyses revealed that approximately 1.2% of all metagenome sequence reads could be assigned to lactococcal bacteriophage or prophage sequences. The metagenome-based reconstruction of the structure of the starter culture community at the level of subspecies or genetic lineages within the subspecies turned out to be impossible. This is due to the high degree of genetic conservation, but at the same time extensive micro-diversification, among the close relative members of the microbial community, which disabled linkage of particular gene-sequences to individual subspecies or lineages. To overcome this, Erkus et al. [4] used different plating media in parallel to recover all bacterial cells from the starter culture, which yielded a collection of hundreds of individual single colony isolates that collectively represented the entire diversity in the starter culture [4]. Following their isolation, a newly developed, high- resolution and high-throughput AFLP-based fingerprinting method was employed [13] to differentiate all single colony isolates at a resolution that exceeds the sub-species level. This genetic fingerprinting approach allowed the identification of 8 distinct genetic lineages of LAB in the undefined starter culture. Interestingly, *Le. mesenteroides *was represented in the culture by a single lineage, whereas *L. lactis *was represented by 7 different lineages. Two of the lactococcal lineages belonged to *L. lactis *subsp. *lactis *while the remaining five were classified as subsp. *cremoris *lineages. The combination of the CFU enumeration with the AFLP-fingerprinting results enabled the determination of the relative abundance of the each of the lineages (Figure 1). The undefined starter culture was found to be dominated (> 90%) by representatives of a single lineage of non-proteolytic *L. lactis *subsp. *cremoris*. The other lineages constituted the remaining 10% of the culture in which the relative abundance of the individual lineages ranged between 0.2% and 2.7%. *Le. mesenteroides *was found to represent approximately 1.8 % of the overall starter culture, which is in good agreement with its estimated relative abundance on the basis of the metagenome analysis. The culture-based procedure was also used to determine the community population dynamics and relative abundances of the identifiable lineages during the entire process of cheese manufacturing (see below).

To obtain more detailed information on the properties of all identified genetic lineages, the full genome sequence was determined of 8 single colony isolates (strains) [4], each representing one of the lineages. This analysis confirmed the taxonomic classification of the lineage-representing strains of each of the lineages as was deduced from AFLP-fingerprinting. Taken together the undefined Gouda cheese starter culture contained: (i) 3 *L. lactis *subsp. *cremoris *lineages carrying the caseinolytic protease (ii) two *L. lactis *subsp. *lactis *biovar. *diacetylactis *lineages that harbour the genes encoding the enzymes and transporters required for co- fermentation of citrate, (iii) 2 non-proteolytic *L. lactis *subsp. *cremoris *lineages and (iv) one citrate degrading, heterofermentative *Le. mesenteroides *lineage. This information confirms that the Ur starter culture is a so-called LD starter, which means that it contains at least *Leuconostoc *sp. (L-type strains) and *L. lactis *subsp. *lactis *biovar. *diacetylactis *(D-type strains). The availability of the complete genome sequence of representative isolates enabled the identification of genetic sequences that are uniquely present in the isolates of a specific lineage, which allowed the design of qPCR approaches for the rapid and reliable molecular quantification of specific lineages in cheese and milk [16].

Although all isolates that were assigned to a particular lineage (based on AFLP-analysis) have a highly similar genomic content, they are not identical. For instance, within each lineage, different plasmid profiles could be identified although not all plasmid profiles were detected in each lineage [4]. This observation implies that extensive, but probably also to some extent lineage-restricted exchange of plasmid DNA takes place within the starter culture community. The observation that not all plasmid profiles were found in all lineages points to incompatibility of certain plasmid pairs [17].

It is already known for a long time that undefined Gouda cheese starter cultures, including the starter culture designated as "Ur", also contain bacteriophages [1,2]. As indicated above, the metagenome of the complex starter culture Ur included the presence of lactococcal bacteriophage assigned sequences. Erkus and co-workers [4] demonstrated the presence of lytic phages in the supernatant of the starter culture, which reflects only a part of the identified bacteriophage sequences within the metagenomic analysis, since the genome sequences of the lineage representing isolates also contained substantial numbers of complete and incomplete prophage sequences. The presence of several lytic phages as well as the many prophages in these starter cultures apparently does not harm their performance in cheese manufacturing, suggesting an inherent functional resilience and stability. Interestingly, using the lytic phages isolated from the culture, a large variation in phage sensitivity was observed, (i) between single colony isolates of a particular genetic lineage and (ii) between the lineages. The highly diverse phage resistance characteristics of the community members is likely to explain the observed functional stability despite the presence of lytic phages, since the consistent presence of resistant variants among the lineage representatives prevents the eradication of an entire lineage from the culture by phage predation [4,18].

Metagenome data sets can be used for the quantification of the relative abundance of the genetic lineages in complex starter cultures. This can be achieved by quantification of the relative read-abundance of sequences assigned to lineage-specific genes that function as lineage specific biomarkers (analogous to their use in qPCR approaches; see above) [16]. However, microbial community profiling on the basis of DNA extracted from environmental samples can be confounded by the presence of DNA derived from dead cells. To overcome this, methods that employ propidium monoazide (PMA) treatment have been developed to enable the selective amplification of DNA derived from the intact and viable fraction of a microbial community [19]. PMA is a membrane-impermeant compound that selectively penetrates cells with compromised membranes, and intercalates into the DNA to which it can be covalently cross-linked through a photo-inducible azide group, resulting in PMA-DNA complexes that strongly inhibit the amplification of the DNA in PCR reactions [19]. Recently, Erkus et al. [16] evaluated the use of PMA for community profiling in ripening Gouda cheese samples. The community dynamics of mixed-strain Gouda cheese starter cultures were determined by metagenomic approaches with and without the application of PMA and compared to the results of lineage specific enumeration by qPCR using lineage specific primers [16]. This study revealed that PMA effectively inhibited the amplification of DNA derived from membrane-compromised cells, and enhanced the selective analysis of the viable population of the cheese starter culture. This enabled the analysis of viable community dynamics that accurately reflected the composition of the viable fraction of the microbial community as it was determined by CFU enumeration. In conclusion, undefined cheese starter cultures, which have a long history of use in the dairy industry and which have evolved from applying the practice of back-slopping, at first sight have a relatively simple community structure (only two co-existing species). However, these cultures were found to possess a substantial degree of genetic variation between the representatives of the species and sub-species present. Three different levels of (genetic) diversity were identified: (i) diversity at the level of genetic lineages that differ in gene content and gene sequence differentiation; (ii) diversity at the level of plasmid content, which appears to be partially independent of the differentiation in genetic lineages; and (iii) diversity at the level of phage resistance within and between genetic lineages. The industrial application of these complex cultures, together with the above mentioned features of the microbial community, make these cultures attractive model systems for studying microbial population dynamics.

### Population dynamics during cheese making

The previous paragraphs outlined a landscape of the structure of the microbial community of complex cheese starter cultures. Understanding the functionality of the concerted action of different lineages in the culture during the process of cheese making, requires information on (i) relative abundances of lineages in the viable fraction of the microbes during cheese manufacturing, and (ii) the gene-expression pattern dynamics during this same process. The reason for this dual information requirement is that milk fermentation and cheese ripening are complex processes in which both the activity of the living microbes as well as the auto-lytic activity of a part of the culture, are important for the ripening process. It is actually the balanced presence of cells that have released their intracellular enzymes (i.e. peptidases, lipases etc.) through lysis and the metabolic activity executed by intact cells within the cheese matrix that determines the formation of the typical cheese aroma compounds [20].

In a standard cheese production and ripening process, only a small proportion (i.e., less that 0.1%) of the LAB present in the cheese at the moment of brining, survives after 6 weeks of ripening [4]. This small fraction of microbes is thought to be responsible for formation of various volatile aroma compounds during ripening such as amino acid-derived aldehydes, carboxylic acids, alcohols and esters [20]. The substantial fraction of the volatiles coming from the breakdown of amino acids [21] and thus originating from the small fraction of live cells, can be explained by the relatively long ripening times used for the production of Gouda type cheeses. To obtain information on the behaviour *in situ *(i.e., in early stages of cheese production) of an individual *L. lactis *strain in a mixed starter culture, a dual selection recombinase-based *in vivo *expression technology (R-IVET) screening system, in combination with a high- throughput cheese-manufacturing protocol was developed. This method allowed the identification and validation of promoter sequences in the *L. lactis *MG1363 genome that were specifically induced during the manufacturing and ripening of cheese [22]. Since *L. lactis *MG1363 does not possess the gene encoding a caseinolytic extracellular proteinase, the strain was applied in conjunction with the complex Gouda cheese starter culture "Bos" [22]. Thereby, the approach of Bachmann and co-workers [22] provided information about the behaviour of an individual strain in the context of a complex community of other starter culture bacteria. During the initial 200 hours of the cheese manufacturing process, 99 promoter elements were identified to be specifically activated in *L. lactis *MG1363. Notably, the genes that were shown to be induced during cheese manufacturing were enriched for genes involved in amino acid metabolism (*cysD, argG, aspC, dapD, hisC, metE2 *and *thrC*) and transport of amino acids and peptides [*ctrA *(*bcaP*), *dppA, brnQ, oppA *and *gltQ*] [22]. This finding implies that cells experience a shortage of amino acids during the early cheese-ripening phase, which is in agreement with the notion that many of these genes identified are predicted to be regulated by the transcriptional repressor CodY [23,24].

It is hypothesized that fluctuations in the relative abundance of members of a complex starter cultures during cheese manufacturing, have a profound effect on functionalities of the starter culture such as acidification rate and/or flavour formation [21]. To find experimental support for this hypothesis, amplified fragment length polymorphism typing of randomly picked single colony isolates obtained at various stages during cheese manufacturing (up to 6 weeks ripened cheese) was used to decipher the population dynamics each of the 8 genetic lineages present in the complex Gouda starter culture [4]. Consistent with previous observations [25], the representatives of the protease negative lineages of *L. lactis *subsp. *cremoris *dominated the microbial community in the first phase of fermentation in which fast acidification of the milk is the key process [4]. This is in agreement with the notion that a relative abundance of 10% or less of proteolytic lactococci in a mixed starter culture is sufficient to supply free peptides and amino acids for an actively fermenting culture [4,25,26]. Theoretically, it is even possible that the 'cheating', protease-negative strains can outcompete protease-positive strains to an extent that leads to extinction of the protease-positive strains [27]. However, Bachmann and co-workers [27] demonstrated experimentally and with modelling approaches that the persistence of the proteolytic trait in a mixed culture is determined by the fraction of the generated peptides that is captured by the protease producing cell before they diffuse away and become equally accessible for the protease- negative cheater population [27].

The addition of salt (brining) to the curd triggers a substantial reduction of the overall community size of viable (colony forming) starter bacteria, which reaches its maximum size after 24 hours of incubation (~10^9 ^cfu/ml) and encompasses a drastic compositional change in the microbial population. The protease negative *L. lactis *subsp. *cremoris *lineage that dominated the initial milk-acidification phase, displayed the fastest decline in viable population after brining, amounting to an almost 6 orders of magnitude reduction in 2 weeks of ripening [4]. Assuming that loss of viability coincides with cell lysis, cells belonging to this lineage are predicted to be the primary suppliers of intracellular enzymes (lipases, peptidases) to the cheese matrix, thereby affecting the initial stages of flavour formation characterized by peptide degradation, and the accumulation of amino acids and fatty acids in the cheese matrix. The role of autolysed LAB in lipolysis has been demonstrated in cheddar cheese [28]. Also secreted esterase was found to impact lipolysis in cheese [29]. Peptide degradation by released peptides plays a role in debittering of the ripening cheese, thereby affecting primarily the taste of the product [30]. The products of peptidolytic and lipolytic activity can serve as substrates for intact, live cells (embedded in the cheese matrix) for the formation of volatile aroma compounds as described above.

During extended cheese ripening, the members of the two citrate degrading *L. lactis *subsp. *lactis *lineages and the *Le. mesenteroides *lineage displayed much better survival characteristics compared to the non-citrate utilizing members of the community [4]. Garcia - Quintans *et al*. [31] demonstrated the induction of expression of the citrate transport system (CitP) in *L. lactis *by lowering of the pH, conditions prevailing at the initial stages of cheese ripening. In *L. lactis*, citrate feeds directly into the pyruvate pool [32] and metabolism of citrate has several important physiological consequences. First of all, citrate metabolisms is linked to the energy status of the cells as it contributes to the proton motive force [33]. In addition, citrate metabolism is linked with pH homeostasis by increasing the pH of the cytoplasm and the medium [32]. Recently, the superior survival of *L. lactis *subsp. *lactis *has been linked to the capacity of these cells to metabolise arginine via the arginine deiminase pathway and to cheese flavour production [34].

Assuming that intact, viable cells are predominantly responsible for the production of volatile aroma compounds in the cheese matrix, it can be concluded that the citrate utilizing bacteria present in complex Gouda cheese starters play a predominant role in the production of the key aroma compounds in Gouda cheese. The superior survival of the citrate degrading *L. lactis *subsp. *lactis *lineages may also be linked to the fact that these strains in general express glutamate decarboxylase [35] that can play a decisive role in survival at low pH [36,37]. The proteolytic *L. lactis *subsp. *cremoris *lineages in the complex starter culture displayed intermediate survival characteristics in the cheese ripening phase [4].

The special role for the only heterofermentative lactic acid bacterium in the culture - *Le. mesenteroides *- can be explained on basis of its metabolic capabilities. It is well established that the production of carbon dioxide by these bacteria as a result of heterofermentative metabolism leads to moderate "eye" formation in the cheese [38]. In addition, recent metatranscriptome analyses during the initial 24 hours of cheese manufacturing using the complex starter culture Ur revealed that approximately 37% of the overall *araT *transcription (encoding aromatic amino transferase) of the complex culture could be assigned to *Le. mesenteroides*, while this lineage constituted only approximately 1% of the overall starter population (data not shown). During the initial acidification phase of cheese manufacturing, *Le. mesenteroides *generally grows slower than the *L. lactis *strains within the starter culture [39]. The relative abundance of *Leuconostoc *sp. versus *Lactococcus *sp. is strongly determined by the temperature regime employed during starter culture propagation. At temperatures above 25°C, *L. lactis *grows substantially faster than *Leuconostoc *sp. [40], whereas at temperatures between 21°C and 25°C a more balanced growth of both genera is observed [39]. During the initial phase of cheese manufacturing, *Le. mesenteroides *is dependent on the caseinolytic *L. lactis *strains for the supply of essential free amino acids or small peptides [41].

The interaction between *Le. mesenteroides *and *L. lactis *in a cheese starter culture is also relevant for aroma formation, since *Le. mesenteroides *can only produce diacetyl and acetoin from citrate at acidic pH [42] and acidification is predominantly driven by lactose fermentation by the lactococci [41]. Interestingly, the importance of *Lc. mesenteroides *for the cheese ripening process is underpinned by the increasing relative abundance of this species during the ripening of cheese [4], which results from its high survival rate compared to the initially dominating *L. lactis *subsp. *cremoris *lineages.

In a recent study, the interactions between *Lactococcus lactis *subsp. *cremoris *SK11 and *Lactobacillus paracasei *ATCC 334 during Cheddar cheese simulation were studied by analysis of the expression of 34 genes common to both bacteria and for eight genes specific to either *L. lactis *subsp. *cremoris *SK11 or *L. paracasei *ATCC 334 [43]. Interestingly, genes linked to stress, protein and peptide degradation as well as carbohydrate metabolism of *L. paracasei *ATCC 334 were overexpressed in mixed culture with *L. lactis *subsp. *cremoris *SK11 during simulation of cheese ripening. Lactococcal genes coding for amino acid metabolism were more expressed during the cheese manufacture simulation, especially in single culture. This study demonstrates complementary functions of starter and non- starter LAB which are relevant for flavor development [43].

In conclusion, the extensive analysis of a complex cheese starter culture performed by Erkus *et al*. [4] illustrates the distinct functional role of the different genetic lineages that co-exist within the starter culture.

### Compositional stability of complex dairy starter cultures

Complex starters originating from traditional propagation by back-slopping are thought to be more stable in composition and function under environmentally fluctuating conditions as compared to defined cultures [1]. However, not much is known about the compositional stability of undefined complex starter cultures used in dairy fermentations. As mentioned in the first paragraph, care has been taken to minimize compositional changes of these industrially important starter cultures. Maintenance of stable culture composition is based on minimizing the numbers of propagation cycles and by keeping sufficient material of the original culture in frozen aliquots. However, it is not clear whether such measures are absolutely required for maintaining the starter culture's composition complexity. Indications of the presence of tight microbe- microbe interactions [4,44] that assure compositional stability suggest a broad tolerance of the culture composition for environmental fluctuations. Erkus and co-workers [4] propagated an undefined Gouda starter culture for 24 days in milk by daily transfer using a 1% (v/v) inoculum size and in total amounting to approximately 155 generations, thereby mimicking a typical back-slopping regime. Using specific qPCR primers targeting all genetic lineages present in the culture, the compositional stability of the culture was monitored during this experiment. This experiment demonstrated that none of the genetic lineages present at the onset of the experiment were lost during back-slopping. This experiment establishes that there is a substantial degree of intrinsic stability of the culture composition. Interestingly, this stability of different genetic lineages of *L. lactis *and *Le. mesenteroides *occurred in the presence of virulent bacteriophages [4]. This so-called phage-carrier state, defined as the continuous presence of virulent bacteriophages throughout the growth of a bacterial culture, is wide-spread in mesophilic mixed-strain starter cultures that predominantly contain lactococci, and is suggested to be related to the high degree of phage insensitivity of these industrial cultures [3]. De Vos [3] explained the phage-carrier state as by a meta- stabile balance between on the one hand curing of plasmids which carry genes encoding phage resistance mechanisms and on the other hand multiplication of phages on sensitive progeny. The complex structure of the cheese starter culture that encompasses 8 co-existing genetic lineages and the high degree of variation of lytic phage sensitivity within each lineage [4] offers an explanation for the observed stability at the level of the microbial community. This explanation is rooted in a process of continuous diversification of a microbial community. The resulting diversity of the community members could prevent the loss of genetic potential from the population as a whole. In line with this hypothesis, heterogeneous phage sensitivity of strains within and between the genetic lineages supports a dynamic process, where phage sweeps eradicate sensitive cells but fail to eradicate entire lineages. The observed persistence of closely related strains within each genetic lineage was in agreement with the density-dependent phage predation model postulated by Rodriguez-Valera and co-workers [18]. The genetic lineages in the Gouda cheese starter culture appeared to be stably present despite continuous phage predation [4], exemplifying that 'kill-the-winner principles' [45] are operational at the strain level, and not at the genetic lineage level.

**Figure 2:**
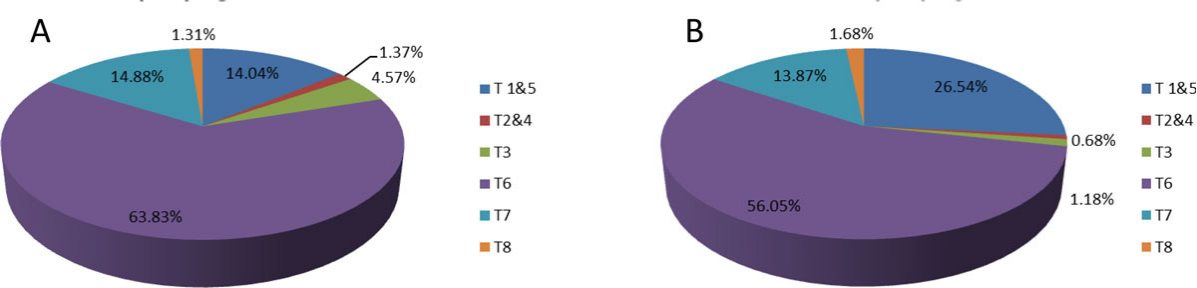
**Composition of sequentially propagated original complex starter culture (panel A) and reconstituted defined starter culture (panel B)**. The cultures were daily propagated in sterile skimmed milk with 1% inoculum for a period 29 days (equivalent to 186 generations) at 20°C. The composition of the cultures at the end-point was measured using lineage specific qPCR primers described in [4]. The colours indicate the genetic lineage and the percentages indicate the measured relative abundance. Data from: De Groof, Wolkers-Rooijackers and Smid, 2013, unpublished results.

Recently, we demonstrated the compositional stability of the undefined Gouda cheese starter culture by comparing the sequentially propagated original cultures with reconstituted cultures containing single strains representing the 8 genetic lineages. After 186 generations of sequential propagation at 20°C, the genetic lineages were found to be present at similar relative abundances in both the original culture and the reconstituted culture (Figure 2). The observation that the reconstituted culture gravitates to the same composition as the original culture demonstrates the inherent compositional stability.

### Functional stability of complex dairy starter cultures

As previously mentioned, the complex dairy cheese starter described by Erkus and co-workers [4] consists of seven genetic lineages of *L. lactis *and a single *Le. mesenteroides *lineage. These genetic lineages can be differentiated from each other on basis of gene content, raising the question whether the presence of all lineages in the starter culture is required for full functionality in terms of flavour formation during cheese ripening. Simplified cultures that contain only a single representative strain of each of the occurring lineages were reconstituted and used for the production of cheese. Cheeses were produced in a high throughput screening model [46] with the original Gouda-cheese starter culture and with a reconstituted culture. Similar aroma profiles were detected during ripening in the cheeses made with the original and the reconstituted culture during ripening, illustrating that complex starter cultures can be reconstructed provided that full knowledge of the composition of the original culture available. However, it can be anticipated that such reconstituted, and simplified, starter cultures are more susceptible to phage predation, since the intrinsic diversity among members of the genetic lineages that is a key-determinant in the phage predation resilience of the original starter culture is abandoned in the reconstituted communities.

Although the presence of the constituting genetic lineages in complex starter cultures seems to be stable under a range of environmental conditions, the relative abundance of the individual lineages may vary as a function of growth environment (temperature, pH) and propagation regime. To illustrate this, the starter culture was propagated by sub- cultivation from the exponential phase of growth versus stationary phase of growth, which affects the relative abundance of the individual lineages [47].

## Conclusions

In depth analysis that combined culture-dependent and culture- independent approaches, provided an unprecedented insight in the complexity of the - at first sight - relatively simple microbial community of an undefined cheese starter culture used for the production of Gouda- type cheeses [4]. The structural complexity of the culture at the level of genetic lineages (below sub-species level) provides an explanation for the compositional stability (robustness) of the culture, which displays substantial resilience even under phage predation pressure. This knowledge can be capitalized in rational reconstitution of starter cultures with intrinsic stability that underlies reliable performance during cheese manufacturing. The genome sequence information of representative strains of the constituent genetic lineages can be used for the construction of stoichiometry based genome scale metabolic models [48-51], which may allow the prediction of the metabolic contributions of the individual members of the microbial community to the metabolic conversions executed by the community as a whole. Preliminary, and relatively simple superimposition of such metabolic models allowed the prediction of potentially beneficial metabolic interaction between *L. lactis *subsp. *lactis *biovar. *diacetylactis *and *Le. mesenteroides *[4]. In this particular example, the metabolic interaction is based on the conversion of glutamate into γ- amino-butyric acid (GABA) and its subsequent secretion by *L. lactis*, which is imported by and converted to succinic acid by *Le. mesenteroides*. Thereby, the conversion of glutamate to succinic acid is predicted to result from the sequential and concerted activities of two members of the microbial community and may deliver benefits for the interacting microbes. The combination of single strain metabolic models into multistrain-models, and the subsequent application of flux balance analysis with the objective to increase biomass yield, is not trivial. Nevertheless, initial attempts in this direction already led to the prediction of several biomass-yield stimulating metabolic interactions between community members that rely on the exchange of specific metabolites. These predicted metabolic interactions could play a role in the inherent robustness of complex starter cultures by creating dependencies between the individual members of the community. Such information is crucial for the rational design of robust composite starter cultures, for which the metabolic models may eventually also predict their flavour formation performance, which could allow designer approaches to deliver new flavour profiles in fermented dairy products.

## Competing interests

The authors declare that they have no competing interests.

## Authors' contributions

EJS drafted the outline and wrote the first versions of all sections. MK contributed to all sections. SA, JW and MS gave important feedback on draft versions of several sections and improved the manuscript by critical reading. OE provided detailed information of compositional changes in the starter culture. All authors read and approved the final manuscript.
